# Optimal antiviral treatment strategies and the effects of resistance

**DOI:** 10.1098/rspb.2010.1469

**Published:** 2010-09-29

**Authors:** Elsa Hansen, Troy Day

**Affiliations:** 1 Department of Mathematics and Statistics, Queen′s University, Jeffery Hall, Kingston, Ontario, Canada K7L 3N6; 2 Department of Biology, Queen′s University, Jeffery Hall, Kingston, Ontario, Canada K7L 3N6

**Keywords:** optimal control, resistance, antiviral, treatment, influenza

## Abstract

Recent pandemic planning has highlighted the importance of understanding the effect that widespread antiviral use will have on the emergence and spread of resistance. A number of recent studies have determined that if resistance to antiviral medication can evolve, then deploying treatment at a less than maximum rate often minimizes the outbreak size. This finding, however, involves the assumption that treatment levels remain constant during the entire outbreak. Using optimal control theory, we address the question of optimal antiviral use by considering a large class of time-varying treatment strategies. We prove that, contrary to previous results, it is always optimal to treat at the maximum rate provided that this treatment occurs at the right time. In general the optimal strategy is to wait some fixed amount of time and then to deploy treatment at the maximum rate for the remainder of the outbreak. We derive analytical conditions that characterize this optimal amount of delay. Our results show that it is optimal to start treatment immediately when one of the following conditions holds: (i) immediate treatment can prevent an outbreak, (ii) the initial pool of susceptibles is small, or (iii) when the maximum possible rate of treatment is low, such that there is little de novo emergence of resistant strains. Finally, we use numerical simulations to verify that the results also hold under more general conditions.

## 1. Introduction

The current interest in influenza pandemics has emphasized the importance of understanding the implications of drug resistance for different types of public health interventions. Recent studies have considered the consequences of drug resistance on the effectiveness of several intervention strategies, including drug prophylaxis and treatment, vaccination, non-drug interventions, as well as combinations of interventions and multi-drug therapy [1–17]. Many of these studies have revealed interesting and unexpected behaviour. In particular, numerous studies have now demonstrated that, with respect to drug treatments, there is sometimes an intermediate optimal level of treatment that minimizes the total outbreak size [8,9,12–14].

This result is somewhat counterintuitive, and arises from a trade-off between the costs and benefits of treating infected individuals. In fact, treatment has three effects on the total outbreak size: (i) treatment is beneficial because it suppresses the spread of the sensitive strain, (ii) treatment is costly because it leads to the de novo appearance of resistant infections, and (iii) treatment is costly because suppression of the sensitive strain frees up susceptible hosts that can then be infected by the resistant strain [9]. The treatment level that minimizes the total outbreak size should thus strike the optimal balance among these three effects.

These findings clearly reveal that the prospect of pathogen evolution during a disease outbreak can have a significant impact on the design of optimal intervention strategies. Nevertheless, it is important to note that most of these results make a key implicit assumption; namely that, whatever treatment level is chosen, it must remain constant during the entire course of the outbreak. An interesting exception is the studies by Moghadas *et al.* [12,13], where numerical examples are used to illustrate that an even smaller total outbreak size can sometimes be obtained by switching from one level of treatment to another once the outbreak is underway.

Given the above collection of results, it is clear that the optimal treatment strategy during an outbreak is not yet completely understood. In this paper, we address this issue through the use of optimal control theory [18–21]. In particular, we consider a model in which we allow for arbitrary, time-varying, treatment strategies during an outbreak, and we derive analytical expressions that characterize the form of the optimal strategy. We prove that, contrary to the implications of previous studies, it is always optimal to use maximum treatment levels *provided that this treatment occurs at the right time*. In general, the optimal strategy is to wait some fixed period of time once the outbreak has begun, and then to deploy treatment at a maximum level. We show that the optimal delay is one that balances the above-mentioned costs and benefits. Finally, we explore extensions of the model to more complex scenarios in order to illustrate the robustness of our conclusions.

## 2. Mathematical model

**Figure 1.**
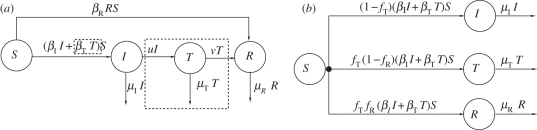
Model structure. (*a*) Schematic for the treatment model that is used to motivate model (2.1). An individual can be susceptible (*S*), infected with a sensitive strain (*I*), infected with a sensitive strain and treated (*T*) or infected with a resistant strain (*R*). The dynamics of the treated class have been enclosed in dashed boxes to emphasize the difference between this figure and the equations for model (2.1). All analytic results are derived using model (2.1), which does not include the dynamics of the treated class. (*b*) Schematic for the detailed model. Using numerical simulations, the analytic results for model (2.1) have been extended to this more detailed model that includes the dynamics of the treated class.

The model, depicted in figure 1*a*, describes the basic transmission dynamics of an infectious disease and includes the effects of treatment, as well as the presence of a strain resistant to treatment. An individual can be susceptible (*S*), infected with a sensitive strain (*I*), infected with a sensitive strain and treated (*T*) or infected with a resistant strain (*R*). Susceptible individuals become infected with the sensitive strain through contact with individuals in the *I* and *T* compartments (with contact parameters *β*_I_ and *β*_T_, respectively), and become infected with the resistant strain through contact with individuals in the *R* compartment (with contact parameter *β*_R_). Individuals infected with the sensitive strain can move into the treated class at some (time-varying) *per capita* rate, *u*(t), where *u* is the treatment strategy of interest. Treated individuals can develop resistance at some constant *per capita* rate, *ν*, and finally, individuals in each of the three infected classes can leave the system, through either death or recovery with immunity, at constant *per capita* rates *μ*_I_, *μ*_T_ and *μ*_R_ (also see electronic supplementary material, §1).

The objective of the analysis is to determine the optimal time-varying treatment strategy, *u*, that minimizes the total attack ratio (i.e. minimizes the attack ratio of the sensitive strain plus the attack ratio of the resistant strain), given by

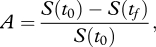

which is equivalent to minimizing the total outbreak size (and to maximizing the final number of susceptibles). The quantities *t*_0_ and *t*_*f*_ are the start and end times of the outbreak, respectively. We say that the outbreak has ended as soon as the total number of all disease-transmitting individuals is equal to 1. In other words, for the model depicted in figure 1*a*, *t*_*f*_ = min{t| *I*(*t*) + *T*(*t*) + *R*(*t*) ≤ 1}. By defining *t*_*f*_ in this way, we avoid the situation where an unrealistically small number of infecteds (namely less than one infected) initiates an outbreak. The rational for this definition of *t*_*f*_ is further detailed in electronic supplementary material, §2 and in Hansen & Day [22].

The treatment level, *u*, can vary through time, and can take on any value from 0 to some maximum value, *u*_max_. The upper bound, *u*_max_, reflects the fact that, regardless of how much we strive to increase the rate of treatment, it will typically be unavoidable that infected individuals spend some amount of time circulating in the population before they are treated. This will occur because of constraints on treatment delivery (e.g. having a finite number of health professionals available to deliver treatment), as well as constraints imposed by the biology of the disease (e.g. if there is an asymptomatic stage).

The above model is qualitatively similar to those examined by Handel *et al.* [8], Lipsitch *et al.* [9], Moghadas *et al.* [13] and Qiu & Feng [14], but we make a further pair of simplifying assumptions to facilitate mathematical analysis: (i) that treatment is perfectly effective, meaning that essentially no transmission occurs from treated individuals, and (ii) that the time dynamics of the treated class are fast relative to those of the other classes. In this case, we can use the quasi-equilibrium value of *T* to simplify the model (electronic supplementary material, §1). This simplification removes the highlighted elements of the flow diagram in figure 1*a*, and yields the following equations:
2.1
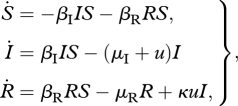

where *κ* denotes the probability that a treated individual develops resistance.

Although the above simplifying assumptions might not be particularly realistic for some situations, our primary aim is to develop a broad conceptual understanding of the optimal treatment strategy during an outbreak. Moreover, these assumptions do not appear to qualitatively alter the results, as evidenced by the numerical simulations presented later. In fact, these simplifications primarily accentuate the three key effects of treatment that were discussed in §1. First, model (2.1) assumes that treatment completely suppresses the spread of the sensitive strain and therefore it maximizes the first effect of treatment (i.e. the suppression of sensitive infections). Second, model (2.1) assumes that, if de novo resistance appears at all, it arises immediately upon treatment because the dynamics of the treated class are assumed to be fast relative to the other classes. Third, model (2.1) also removes the impact of treated individuals on the depletion of susceptible hosts, therefore maximizing the third effect of treatment (i.e. the freeing-up of susceptible hosts for infection by resistant strains).

## 3. Results

To facilitate the understanding of our results, we first define some important quantities and briefly describe some of the basic features of the optimal treatment strategy. We then give a complete characterization of the optimal treatment strategy in two steps. First, we consider a special case (situation 1) in which we can use standard techniques from calculus to gain an intuition for the results. Second, we characterize the optimal treatment strategy in general (situation 2). Analysis of the latter case requires techniques from optimal control theory, and these details are presented in the electronic supplementary material.

The optimal treatment strategy for model (2.1) is characterized by the following three quantities:

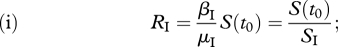


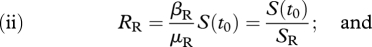


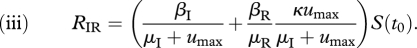



Here, *R*_I_ and *R*_R_ are the basic reproduction numbers of the sensitive and resistant strain, respectively [23–25]. The quantities *S*_I_ = *μ*_I_/*β*_I_ and *S*_R_ = *μ*_R_/*β*_R_ represent threshold parameters, and denote the number of susceptibles above which a sensitive and resistant outbreak can occur, respectively. To understand the constant *R*_IR_, consider the possible paths that a new sensitive infection can take. If the population is receiving treatment (i.e. *u* ≠ 0), then an initially sensitive infection may develop resistance and so some initially sensitive infections will generate both sensitive and resistant infections during their lifetime. The constant *R*_IR_ can be interpreted as the total expected number of secondary infections (both sensitive and resistant) caused by an individual initially infected with the sensitive strain in a wholly susceptible population that is receiving maximum treatment (i.e. *u* ≡ *u*_max_). Specifically, the first term represents the number of infections generated while infected with the sensitive strain, and the second term represents the probability of developing resistance, multiplied by the number of infections generated while infected with this newly evolved resistant strain.

A few more general observations will be helpful before stating the results. First, *R*_IR_ can be expressed as *R*_IR_ = *R*_I_ (1 − *χ*) + *χκR*_R_, where *χ* = *u*_max_/(*μ*_I_ + *u*_max_) is the probability of an infected individual receiving treatment when the treatment effort is *u* ≡ *u*_max_. This expresses *R*_IR_ as a mixture of the two reproduction numbers, *R*_I_ and *R*_R_. Second, because the number of susceptibles always decreases through time, it is sometimes easier to express the optimal treatment strategy as a function of the size of the susceptible pool rather than as a function of time *per se*. This is also more useful from a health policy point of view because ‘number of susceptibles' is a much more meaningful (and more easily measured) quantity than ‘time since start of outbreak’. Thus, although in the sequel we will discuss ‘treatment start time’ and ‘delay’, these will sometimes be measured in terms of the corresponding number of susceptible individuals. For reference all symbols have been defined in table 1.

### (a) The optimal treatment strategy

*The optimal treatment strategy is to delay treatment until some fixed time* τ* (*possibly* τ* = *t*_0_) *and then to treat maximally for the remainder of the epidemic.* Thus, higher treatment levels are always better provided that the onset of treatment is delayed by the optimal amount.

### (b) The optimal amount of delay

To simplify the conditions for the optimal delay, suppose that *R*_I_ ≥ *R*_R_ (the resistant strain suffers a fitness cost in the absence of treatment) and *κ* < 1 (not all treated infections develop resistance; the complete analysis is presented in the electronic supplementary material). In this case, the optimal delay can be characterized in terms of a critical number of susceptible hosts, defined by
2.1
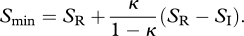



The optimal delay is achieved by balancing the effect that delay has on the sensitive and resistant outbreaks, and this is best understood by considering two separate situations: (1) the maximum treatment level is very large (*u*_max_ → ∞) and (2) the maximum treatment level is not very large (*u*_max_ is finite).

*Situation 1*. If the maximum treatment level is very large, then some insight into the optimal treatment strategy can be gained using basic calculus. In particular, if the maximum level of treatment is used, then the onset of treatment effectively ends the sensitive epidemic and initiates the start of the resistant epidemic. This is because at the onset of treatment, all sensitive-infected individuals enter either the removed compartment (i.e. they are treated and no longer transmitting the infection) or the resistant compartment (i.e. treatment causes them to immediately develop resistance). Therefore, before treatment begins, model (2.1) simplifies to
3.1
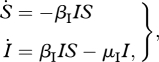

with initial conditions (*S*(*t*_0_),*I*(*t*_0_)). Once treatment begins at time *t* = *τ*, all of the sensitive-infected individuals either (i) immediately become resistant or (ii) immediately stop transmitting the infection. Thus, for *t* > *τ*, model (2.1) simplifies to
3.2
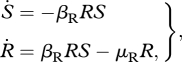

with (*S*(*τ*),*R*(*τ*)) = (*S*(*τ*),*κI*(*τ*)). Hence, in this special case, determining the optimal treatment strategy amounts to determining the optimal time to switch from model (3.1) to model (3.2).

Integrating and rearranging the equations for model (3.1) and model (3.2) give (see electronic supplementary material, §3, for details):
3.3
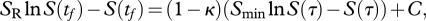

where *C* = *κ*(*S*_I_ ln*S*(*t*_0_) − *S*(*t*_0_) − *I*(*t*_0_)) + *R*(*t*_*f*_). Now since, by definition, the outbreak ends as soon as the total number of infecteds is less than or equal to 1, there are two possibilities with respect to the optimal delay.

(A) *Treating immediately can prevent the outbreak*: if *κI*(*t*_0_) < 1, then starting treatment at *t*_0_ prevents the outbreak (i.e. *R*(*τ*) = *κI*(*τ*) < 1). Clearly, this is the best possible scenario, and therefore τ* = *t*_0_. Notice that this situation is closely related to the idea of ‘containment’ discussed in Lipsitch *et al.* [9].

(B) *Treating immediately cannot prevent the outbreak*: if the outbreak cannot be prevented through treatment, then any treatment strategy will result in *S*(*t*_*f*_) ≤ *S*_R_ (i.e. the outbreak will not end until the pool of susceptibles is diminished below that necessary to sustain an resistant outbreak). Therefore, the treatment start time that maximizes the left-hand side of equation (3.3) will also maximize *S*(*t*_*f*_) (and hence minimize the total attack ratio). In other words, it is optimal to delay treatment until the number of susceptible hosts has declined to

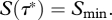

If the initial number of susceptible hosts is smaller than this threshold, then treatment should begin immediately.

*Situation 2*. If the maximum treatment level is bounded, then there will be some temporal overlap in the sensitive and resistant epidemics once treatment has been initiated. In this situation, Pontryagin's maximum principle can be used to determine the optimal (time-varying) treatment strategy (see electronic supplementary material, §4, for details). There are, again, two possibilities with respect to the optimal delay.

(A′) *Treating immediately can prevent the outbreak*: if starting treatment at *t*_0_ prevents the outbreak from occurring, then this is clearly the best possible scenario, and so τ* = *t*_0_. As with situation 1, this situation is closely related to the idea of ‘containment’ discussed in Lipsitch *et al.* [9] and is contingent on how *t*_*f*_ is defined (i.e. for model (2.1) we have that *t*_*f*_ = min{*t*|*I*(*t*) + *R*(*t*) ≤ 1}).

(B′) *Treating immediately cannot prevent the outbreak*: if scenario (A′) does not hold, then it may be optimal to delay the onset of treatment. In particular, the threshold number of susceptibles *S*_min_ now provides an upper bound on the optimal delay. Thus, if the initial number of susceptible hosts is smaller than this threshold, then treatment should begin immediately. On the other hand, if *S*(*t*_0_) is initially larger than *S*_min_, then the optimal delay is no larger than the time it takes for the number of susceptible hosts to decline to *S*_min_. There is one other possibility, however, that overrides these conditions: if *R*_IR_ > *R*_R_, then it is optimal to start treatment immediately.

Note that there are two major differences between the results for situation 1 and situation 2. First, when maximum treatment levels are bounded (situation 2), the critical threshold, *S*_min_, provides only an upper bound for the optimal delay. Second, in situation 2, there is an additional condition under which immediate treatment is optimal; namely, when *R*_IR_ > *R*_R_. Intuitively, if de novo resistance has a negligible effect on the outbreak, then it seems reasonable that treatment should proceed as though there is no de novo resistance. The condition that treatment should begin immediately if *R*_IR_ > *R*_R_ actually highlights this intuition and emphasizes that the quantity that measures the effect of de novo resistance is the rate of de novo resistance (*κuI*) and not the probability of de novo resistance (*κ*). This can be seen by rearranging *R*_IR_ > *R*_R_ and multiplying both sides by *κI* to produce a relationship involving the rate of de novo resistance:
3.4
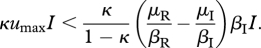

Hence, if the rate of de novo resistance is small, then treatment should proceed as though there is only a treatment-sensitive strain (i.e. if equation (3.4) holds then treatment should start immediately).

### (c) Interpretation

We have shown that, for a simple model which includes the evolution of resistance, the optimal treatment strategy is to delay treatment for a specific amount of time (possibly no time) and then to treat with maximum effort for the remainder of the epidemic. If an outbreak is unavoidable, the optimal amount of delay is determined by balancing the benefits and costs of delay, and this trade-off can be best understood by again considering the case when the maximum treatment level is very large.

When the maximum treatment level is very large (i.e. situation 1), the marginal change in the attack ratio that comes from increasing the amount of delay before treatment is (see electronic supplementary material, §3, for details):
3.5
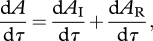

3.6
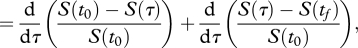

3.7
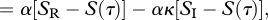

where *A*, *A*_I_, *A*_R_ denote the total, sensitive and resistant attack ratios, respectively, *τ* is the treatment start time and *α* = *S*(*t*_*f*_)*β*_I_*I*(*τ*)/[*S*(*t*_0_)(*S*_R_ − *S*(*t*_*f*_))] > 0.

**Figure 2.**
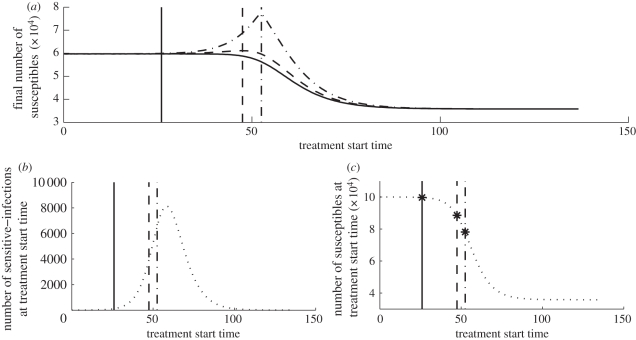
Effect of treatment start time on total attack rate (situation 1B). The vertical lines indicate the optimal treatment start time τ* for a large *κ* (solid line), an intermediate *κ* (dashed line) and a small *κ* (dotted-dashed line). (*a*) The final number of susceptibles as a function of treatment start time for a large *κ* (*κ* = 0.58; solid curve), an intermediate *κ* (*κ* = 0.4; dashed curve) and a small *κ* (*κ* = 0.001; dotted-dashed curve). (*b*) The number of sensitive infections as a function of treatment start time, *τ*. The total attack rate is decreased by decreasing the number of sensitive infections at the treatment start time (*I*(*τ*)) and by decreasing the number of susceptibles at the treatment start time (*S*(*τ*)) (equation (3.7)). As *κ* increases, the effect of decreasing *I*(*τ*) becomes more important than decreasing *S*(*τ*); therefore, τ* decreases as *κ* increases (from left to right, the order of the vertical lines is solid, dashed and dot-dashed). (*c*) The number of susceptibles as a function of treatment start time. As *κ* increases, *S*(τ*) increases. Also, the points *S* = *S*_min_ are indicated by ‘star’ markers and coincide with *S*(τ*). Parameters are *R*_I_ = 1.6, *R*_R_ = 0.8*R*_I_ and *μ*_I_ = *μ*_R_ = 1/3.3.

Equation (3.5) emphasizes that the total attack ratio can be decomposed into a sensitive attack ratio and a resistant attack ratio. Furthermore, since for situation 1 the sensitive and resistant outbreaks are temporally separated (because the resistant strain emerges only after treatment begins), the sensitive attack ratio includes all new infections that occur before the treatment start time *τ*, and the resistant attack ratio includes all new infections that occur after the treatment start time *τ* (see equation (3.6)). The two terms in equation (3.7) represent the benefit and cost of delay, respectively. The first term represents the benefit of a delay that comes from a reduction in the size of the susceptible pool at the start of the resistant-strain epidemic. From the perspective of this benefit term only, it is best to delay treatment until *S*(*τ*) = *S*_R_, at which point there are no longer enough susceptible individuals to sustain a resistant outbreak. The second term represents the cost of delay that comes from an increase in the number of de novo resistant infections once treatment begins. From the perspective of this cost term only, it is best to treat immediately because waiting longer results in a larger pool of infected individuals, which then have the potential to develop de novo resistance once treatment begins. The optimal amount of delay precisely balances these two effects, and this can be readily interpreted graphically. Figure 2*a* shows the final number of susceptibles as a function of treatment start time for different values of *κ*. As *κ* increases, the importance of the second term in equation (3.7) increases and correspondingly the number of infecteds at the optimal treatment start time decreases (figure 2*b*), while the number of susceptibles at the optimal treatment start time increases (figure 2*c*).

### (d) Numerical results for more detailed models

Although the analytic expressions presented above are derived using a simple model, numerical simulations suggest that these results hold more generally. To illustrate this, we use numerical simulations to compare the relationship between treatment start time and attack ratio for our simple model and for the more detailed model in Lipsitch *et al.* [9]. Figure 1*b* depicts the treatment model used in Lipsitch *et al.* [9] and the corresponding system equations are provided in electronic supplementary material, §5.

To begin, numerical results show that both models exhibit two key features: (i) if the treatment level is constrained to be constant throughout the entire epidemic (as has been the case in most previously published analyses), then there is an intermediate optimal level of treatment, and (ii) if treatment is delayed by the appropriate amount, then maximum treatment is always optimal (electronic supplementary material, figures 1 and 2). The fact that delaying treatment can be better than constant treatment in both models explains why Lipsitch *et al.* [9], Moghadas *et al.* [13], Handel *et al.* [8] and Qiu & Feng [14] observed an intermediate optimal constant treatment level.

**Figure 3.**
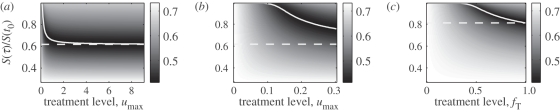
Total attack ratio versus treatment level and number of susceptibles at treatment start time (normalized by *S*(*t*_0_)). The white solid curves show the number of susceptibles at the optimal treatment start time provided a resistant epidemic occurs. The white dashed horizontal lines indicate the number of susceptibles at the optimal treatment start time when the maximum possible treatment level is very large (i.e. when *u*_max_ is unbounded for the simple model and when *f*_T_ = 1 for the detailed model). The white dashed horizontal lines in (*a*) and (*b*) were computed analytically; all other curves were computed numerically. It is important to emphasize that this figure was generated by assuming that an outbreak occurs. Indeed, for this specific choice of parameters, electronic supplementary material, figure 3, illustrates that for *u*_max_ > 0.25 and *f*_T_ > 0.8 treating immediately will prevent an outbreak and so treating immediately is optimal. (*a*) Figure produced using simple model. (*b*) Magnified version of (*a*). (*c*) Figure produced using detailed model. Parameters are *R*_R_ = 0.9*R*_I_, *μ*_1_ = *μ*_2_ = *μ*_3_ = 1/3.3, *κ* = 0.0066, *f*_r_ = 0.002.

A comparison of the panels of figure 3 shows that the general relationship between the treatment level, the attack ratio and the number of susceptible hosts at the start of treatment is qualitatively similar for both models. Furthermore, figure 3*a* shows that for the simple model, if an outbreak occurs, then *S*(τ*) approaches *S*_min_ as the treatment level increases (i.e. the solid curve approaches the horizontal dashed line). In other words, the optimal solution for the case when *u*_max_ is very large provides a bound for the case when *u*_max_ is finite. A similar statement is true for the more detailed model. In the more detailed model, *f*_T_ denotes the fraction of infections that are treated, and so *f*_T_ = 1 is the analogous case to *u*_max_ being unbounded (i.e. all infected individuals are treated). Using *S*_min,c_ to denote the number of susceptibles at the optimal treatment start time when *f*_T_ = 1, figure 3*c* shows that, for the detailed model, *S*(τ*) decreases as the treatment level increases, with *S*_min,c_ providing a bound for all other values of *f*_T_.

**Table 1.** Table of symbols.

symbol	brief definition
*S*(*t*)	number of susceptibles at time *t*
*I*(*t*)	number of individuals infected with the treatment-sensitive strain at time *t*
*T*(*t*)	number of treated individuals at time *t*
*R*(*t*)	number of individuals infected with the treatment-resistant strain at time *t*
*β*_I_	contact parameter for the sensitive strain
*β*_T_	contact parameter for the treated strain (*β*_T_ = 0 for the simple model)
*β*_R_	contact parameter for the resistant strain
*μ*_I_	*per capita* rate of death or recovery with immunity for the sensitive-infected class
*μ*_T_	*per capita* rate of death or recovery with immunity for the treated class
*μ*_R_	*per capita* rate of death or recovery with immunity for the resistant-infected class
*u*(*t*)	*per capita* rate of treatment for the sensitive-infected class at time *t*
*v*	*per capita* rate of developing resistance for the treated class (applies to model in figure 1*a*)
*κ*	probability that treated individual develops resistance (applies to simple model, see equation (2.1))
*t*_0_	the outbreak start time
*t*_f_	the outbreak end time
*u*_max_	upper bound for *per capita* treatment rate *u*
*R*_I_	the basic reproduction number for the sensitive strain
*R*_R_	the basic reproduction number for the resistant strain
*R*_IR_	the expected number of secondary infections (both sensitive and resistant) caused by an individual initially infected with the sensitive strain, in a wholly susceptible population that is receiving maximum treatment (i.e. *u* ≡ *u*_max_)
*S*_I_	the number of susceptibles above which a sensitive outbreak can occur
*S*_R_	the number of susceptibles above which a resistant outbreak can occur
*χ*	the probability that an infected individual receives treatment (assuming *u* ≡ *u*_max_)
*τ*	an arbitrary treatment start time
τ*	the optimal treatment start time
*f*_T_	the fraction of infected individuals that receives treatment (applies to detailed model, see figure 1*b*)
*S*_min_	the number of susceptibles at the optimal treatment start time assuming that an outbreak occurs and *u*_max_ is unbounded (applies to simple model)
*S*_min,c_	the number of susceptibles at the optimal treatment start time assuming that an outbreak occurs and *f*_T_ = 1 (applies to detailed model)
*A*	the total attack ratio

Both models also demonstrate that it is possible to avoid an outbreak by starting treatment immediately at sufficiently high treatment levels (electronic supplementary material, figure 3). Furthermore, if the initial pool of susceptibles is small enough, then it is always best to start treatment immediately (electronic supplementary material, figure 4). For the simple model, ‘small enough’ is measured relative to the number of susceptibles at the optimal treatment start time when *u*_max_ is unbounded. Namely, if *S*(*t*_0_) < *S*_min_, then τ* = *t*_0_. Similarly, for the more detailed model, electronic supplementary material, figure 4*b*, suggests that if *S*(*t*_0_) < *S*_min,c_ then again it is optimal to start treatment immediately.

Finally, recall that for the simple model if *R*_IR_ > *R*_R_, then it is optimal to start treatment immediately. Using the biological interpretation of *R*_IR_, namely that *R*_IR_ is the expected total number of infections caused by an individual initially infected with the treatment-sensitive strain, we can define an analogous quantity for the detailed model (see electronic supplementary material, text S1, for details). Electronic supplementary material, figures 5 and 6 show that the relationship between *R*_R_, *R*_IR_ and the optimal treatment start time is similar for both the simple and detailed models. This result is particularly interesting because it emphasizes that (i) using a simple model can highlight relationships in more detailed models that may otherwise be undetected, and (ii) the quantity *R*_IR_ is of general importance and not just an artefact of our simple model.

## 4. Discussion

Perhaps the most salient feature of previous analyses is that the best treatment somehow balances the effects of the sensitive and resistant epidemics. This trade-off, which is very clearly explained in the discussion found in Lipsitch *et al.* [9], is also highlighted in our model. The difference is in the way this trade-off is resolved. In Lipsitch *et al.* [9], this trade-off is used to explain why an intermediate level of constant treatment is better than a high level of constant treatment. Conversely, we have shown that the optimal treatment balances this trade-off by delaying the onset of treatment instead of capping the maximum level of treatment. By allowing the treatment strategy to vary in time, we have shown that higher levels of treatment are always better, provided they are started at the appropriate time. Furthermore, when the maximum possible treatment level is very large, our analysis shows that the optimal treatment strategy is the one that switches from a sensitive outbreak to a resistant outbreak precisely when the number of susceptible hosts has decreased to the value given by *S*_min_.

We have examined two types of model, a simple model that does not explicitly include the dynamics of the treatment class and a more detailed model that does include treatment dynamics. For the simple model, we showed, using analytic calculations, that the optimal treatment strategy is ‘off–on’ (i.e. delay treatment for a certain amount of time—possibly no time—and then treat maximally for the remainder of the outbreak). It is important to emphasize that for model (2.1) this off–on strategy is indeed optimal in the sense that any other, more complicated, strategy that involves intermediate levels of treatment will not result in a lower attack ratio. Conversely, for the more detailed model, we have only shown (via numerical simulations) that an off–on strategy can outperform a ‘constant’ strategy. Thus, although it seems likely that the off–on strategy is optimal for the more detailed model as well it remains an open problem to explicitly prove that this is indeed the case. Furthermore, although an off–on strategy can be better than a constant strategy, policy makers still face the ethical dilemma of choosing between the rights of individuals to receive treatment and the benefit to the population of delaying treatment.

Given the ethical problems associated with implementing the optimal treatment strategy when it involves a delay, it is useful to clearly delineate the conditions under which implementing treatment immediately is actually optimal. It is always optimal to start treatment immediately if one of three conditions holds: (i) immediate treatment can prevent an outbreak, (ii) the initial pool of susceptibles is small (i.e. *S*(*t*_0_) < *S*_min_ for the simple model and *S*(*t*_0_) < *S*_min,c_ for the detailed model), or (iii) the rate of de novo resistance is small (i.e. *R*_IR_ > *R*_R_). Each of these three situations have relevance to public health policy.

Condition (i) underscores that our focus has been to minimize the total outbreak size, and so in this context preventing an outbreak is the best possible outcome. This outcome, however, may not be desirable if there is a high probability of a second epidemic occurring at a later time. For example, although treating immediately at a very high level may minimize the total attack ratio, this will result in *S*(*t*_*f*_) > *S*_R_ > *S*_I_. As a result, the population will be susceptible to future sensitive and resistant outbreaks. Alternatively, if a treatment strategy is chosen to ensure that a resistant epidemic occurs, then the final number of susceptibles will be small enough to prevent future resistant outbreaks (and the severity of a possible future sensitive outbreak will be significantly decreased). Antia *et al.* [26] discusses these issues in the context of a single-strain epidemic. Interestingly, if delay is optimal, then, up to a point, increasing *u*_max_ will increase the optimal amount of delay. However, if *u*_max_ can be increased sufficiently, then treating immediately will effectively prevent the outbreak, and this then becomes the optimal strategy. If this can occur, then policy makers will have to balance the benefits of treating immediately (and therefore preventing the current outbreak) with the benefits of delaying treatment (and perhaps minimizing the effects of a second outbreak).

Condition (ii) highlights that the optimal treatment strategy depends on the size of the target community. Essentially, if a community is small enough, then treatment should start immediately. This observation leads to an interesting hypothesis for combined vaccination–treatment policies. Namely, since vaccination decreases the susceptible population, low vaccination levels make it more likely that treatment should be delayed. This proposed relationship is surprising since, naively, it seems reasonable to expect that if fewer individuals are vaccinated, then it would be better to start treatment early in order to reduce spread. Nevertheless, our results suggest that this intuition is, in fact, incorrect.

Condition (iii) emphasizes that if the probability of receiving treatment is low, then it is more likely that treatment should start immediately. More concretely, by expressing *R*_IR_ as a linear combination of the two reproduction numbers *R*_I_ and *R*_R_, we can rewrite *R*_IR_ > *R*_R_ as

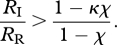

Therefore, if *R*_I_/*R*_R_ > (1 − *κχ*)/(1 − *χ*), it is optimal to start treatment immediately and if *R*_I_/*R*_R_ ≤ (1 − *κχ*)/(1 − *χ*), then it may be best to delay treatment. For any specific values of *R*_I_, *R*_R_ and *κ*, we see that as the probability of treating an infected individual, *χ*, decreases, it becomes more likely that starting treatment immediately is best. Intuitively, this guideline makes sense since if treatment is unlikely to reach many infected individuals, then it makes sense to start administering treatment as soon as possible in order to reach more individuals.

Since multiple intervention measures are often used to control a disease, an important next step is to consider a model that includes other intervention measures, in addition to treatment [4,5,7,10]. Some models similar to our model that have included multi-intervention strategies are the treatment–vaccination models in Ferguson *et al.* [6] and Qiu & Feng [14], and the treatment-prophylaxis model in Lipsitch *et al.* [9]. Interestingly, a number of studies that consider multi-intervention strategies also exhibit optimal constant intervention levels. This suggests that considering multi-intervention strategies that vary in time may be very informative as well.
